# In Vitro CRISPR/Cas9 Transfection and Gene-Editing Mediated by Multivalent Cationic Liposome–DNA Complexes

**DOI:** 10.3390/pharmaceutics14051087

**Published:** 2022-05-19

**Authors:** Diana A. Sousa, Ricardo Gaspar, Celso J. O. Ferreira, Fátima Baltazar, Ligia R. Rodrigues, Bruno F. B. Silva

**Affiliations:** 1 CEB—Centre of Biological Engineering, University of Minho, Campus de Gualtar, 4710-057 Braga, Portugal; diana.sousa@ceb.uminho.pt; 2 INL—International Iberian Nanotechnology Laboratory, Av. Mestre José Veiga, 4715-330 Braga, Portugal; ricardo.gaspar@inl.int (R.G.); celso.ferreira@inl.int (C.J.O.F.); 3 LABBELS—Associate Laboratory, 4710-057 Braga, Portugal; 4 CF-UM-UP, Department of Physics, University of Minho, Campus de Gualtar, 4710-057 Braga, Portugal; 5 Life and Health Sciences Research Institute (ICVS), School of Medicine, University of Minho, 4710-057 Braga, Portugal; fbaltazar@med.uminho.pt; 6 ICVS/3B’s—PT Government Associate Laboratory, 4805-017 Guimarães, Portugal

**Keywords:** CRISPR, Cas9, gene knockout, CL-DNA, lipoplex, plasmid, gene delivery, multivalent cationic lipids, MVL5

## Abstract

Clustered regularly interspaced short palindromic repeats (CRISPR) and CRISPR-associated nuclease 9 (Cas9) gene-editing offers exciting new therapeutic possibilities for disease treatment with a genetic etiology such as cancer, cardiovascular, neuronal, and immune disorders. However, its clinical translation is being hampered by the lack of safe, versatile, and effective nonviral delivery systems. Herein we report on the preparation and application of two cationic liposome–DNA systems (i.e., lipoplexes) for CRISPR/Cas9 gene delivery. For that purpose, two types of cationic lipids are used (DOTAP, monovalent, and MVL5, multivalent with +5*e* nominal charge), along with three types of helper lipids (DOPC, DOPE, and monoolein (GMO)). We demonstrated that plasmids encoding Cas9 and single-guide RNA (sgRNA), which are typically hard to transfect due to their large size (>9 kb), can be successfully transfected into HEK 293T cells via MVL5-based lipoplexes. In contrast, DOTAP-based lipoplexes resulted in very low transfection rates. MVL5-based lipoplexes presented the ability to escape from lysosomes, which may explain the superior transfection efficiency. Regarding gene editing, MVL5-based lipoplexes achieved promising GFP knockout levels, reaching rates of knockout superior to 35% for charge ratios (+/−) of 10. Despite the knockout efficiency being comparable to that of Lipofectamine 3000^®^ commercial reagent, the non-specific gene knockout is more pronounced in MVL5-based formulations, probably resulting from the considerable cytotoxicity of these formulations. Altogether, these results show that multivalent lipid-based lipoplexes are promising CRISPR/Cas9 plasmid delivery vehicles, which by further optimization and functionalization may become suitable in vivo delivery systems.

## 1. Introduction

The discovery of clustered regularly interspaced short palindromic repeats (CRISPR) and CRISPR-associated nuclease 9 (Cas9) has opened new possibilities to knockout or repair genes, revolutionizing the concept of gene therapy and promoting new exciting therapeutic possibilities [1,2]. CRISPR/Cas9 is a two-component system composed of Cas9, an RNA-guided endonuclease capable of cleaving double-stranded DNA, and a 20-nucleotide target-specific sequence specified by single-guide RNA (sgRNA), which directs the Cas9 to a target site for DNA cleavage. The cleavages can be repaired by the nonhomologous end-joining (NHEJ) or homology-directed repair (HDR) pathways [3,4]. The NHEJ is associated with variable sizes of insertions or deletions (indels) that interrupt the expression of target genes by the frame shift occurring in the coding regions. This typically causes gene disruption and knockout. In contrast, gene knock-in and precise gene corrections can be achieved by HDR using a donor DNA template [5]. These features make the CRISPR/Cas9 system an exciting therapeutic possibility for treating, preventing, or curing a wide range of diseases [6]. 

Despite this obvious promise, delivery of CRISPR/Cas9 is one of the major technical concerns limiting the therapeutic application of this technology [7]. This system can be delivered in the form of a DNA plasmid encoding both the Cas9 protein and sgRNA [8], or it may be delivered in the form of Cas9 mRNA [9,10] or native Cas9 protein [11], in which both cases the sgRNA needs to be co-delivered as well. Viral vectors, such as lentiviral vectors, adenoviral vectors, and adeno-associated virus (AAV) vectors have been used most often for the delivery of Cas9/sgRNA-encoded plasmids for CRISPR therapeutics, showing excellent gene transfection efficiency (TE). However, viral expression results in immunogenic responses, long-term expression, and off-target effects [12,13]. Moreover, a large fraction of the human population has pre-existing immunity to AAV, making them ineligible for AAV-based therapies [14]. Therefore, a panel of nonviral vectors is being developed to address the limitations of viral-based vectors and improve genome editing for both in vitro and in vivo applications [8,10,15,16]. 

Cationic liposomes (CLs) are among the most promising vectors for delivering nucleic acids to cells, including DNA plasmids, mRNA, and siRNA [17,18,19,20,21,22,23,24,25,26]. Their cationic charge mediates strong electrostatic interactions with the negative charges of nucleic acids, giving rise to the formation of CL-NA complexes (often called lipoplexes) [23,25,26,27]. These complexes adopt internal nanostructures of lamellar, hexagonal or cubic bicontinuous symmetry, with lipid membranes embedding the nucleic acids [24,25,28,29,30,31]. By manipulation of the cationic-to-anionic charge ratio, CR (+/−), between liposomes and nucleic acids, as well as adjusting the lipid membrane charge density [32,33], level of PEGylation [34,35], and inclusion of stimuli-responsive or targeting functionalization [36,37,38,39,40,41,42], these particles can be made highly efficient. While the efficiency is still not at the level of viral methods, these particles are generally safer, less immunogenic, and simpler to manufacture on larger scales. A related type of lipid nanodelivery system, using ionizable lipids that are cationic only at lower pH [22,43,44], is the base of current mRNA vaccines being used worldwide for COVID-19 prevention [45,46]. In a landmark recent result, an early-stage clinical trial has shown that ionizable lipid nanoparticles encapsulating mRNA-encoded Cas9 and sgRNA targeting the transthyretin amyloidosis gene led to a marked decrease of the misfolded protein in blood, opening excellent prospects for future treatments [47]. 

Besides the recent excitement of RNA-based approaches, co-delivery of two components with significantly different sizes such as Cas9 mRNA (4.5 kb) and sgRNA (0.1 kb), is still challenging [16]. Plasmid DNA, on the other hand, can be engineered to encode both the Cas9 gene and the sgRNA within one plasmid, making it simpler to deliver. However, the large size of Cas9/sgRNA plasmids (9–19 kb) may hinder its effective and efficient intracellular transfection [48,49,50].

In recent years, multivalent cationic lipids have been proposed as a promising strategy to boost the TE of lipid-DNA complexes [33,51,52,53,54]. The use of multivalent cationic lipids (e.g., DOSPA [51] and MVL5 [55]) instead of monovalent ones allows for reaching higher lipid membrane charge densities, which mediate favorable interactions with anionic cell and endosomal membranes, leading to higher transfection [52]. Simultaneously, lipids with a higher valence allow reducing the number of used cationic lipid molecules, which enables a reduction in cytotoxicity, and at the same time allows the inclusion of larger amounts of helper lipids in the membranes, which can also lead to enhanced transfection and gene silencing. Lipoplexes composed of MVL5 and different helper lipids, including monoolein (GMO), 1,2-dioleoyl-sn-glycero-3-phosphocholine (DOPC), and cholesterol, were shown to have superior TE compared to monovalent cationic lipid formulations, with MVL5/GMO complexes also showing superior performance in the presence of serum and in harder-to-transfect human cell lines [53].

In this work, we aimed to study the suitability of multivalent cationic lipid-DNA complexes for delivery and transfection of CRISPR/Cas9 DNA plasmids to in vitro human cells (HEK 293T) and the resulting gene knockout. MVL5, a pentavalent cationic lipid, introduced by Ewert and Safinya in 2002 [55], is now commercially available and was chosen as the multivalent lipid. Besides the charge of the liposome membrane, also its elastic properties and propensity to form non-bilayer structures are thought to be important in facilitating the fusion of lipoplexes with the endosomal membrane and improving transfection [43,56,57]. Hence, three different helper lipids, DOPC, GMO, and 1,2-dioleoyl-sn-glycero-3-phosphoethanolamine (DOPE), were used in addition to MVL5, to produce four different binary lipid formulations (MVL5:DOPC 50:50, MVL5:GMO 50:50, MVL5:DOPE 50:50, and MVL5:DOPE 75:25). While all resulting lipid–DNA complexes are expected to show a lamellar-type nanostructure for the used compositions [33,57], the DOPE and GMO lipids have a higher propensity to form inverted lipid phases, such as reverse hexagonal and, in the case of GMO, also bicontinuous cubic phases [56,57,58]. Hence, by combining a highly-charged lipid with three lipids with different propensities to form non-bilayer structures, we hope to identify a regime of suitable transfection efficiency for the large plasmids containing the Cas9 and sgRNA sequences. For comparison purposes, we used also analogous formulations using the monovalent 2,3-Dioleyloxypropyltrimethylammonium chloride (DOTAP) lipid, which is one of the most used lipids in transfection, as well as the commercial Lipofectamine 3000^®^.

**Figure 1:**
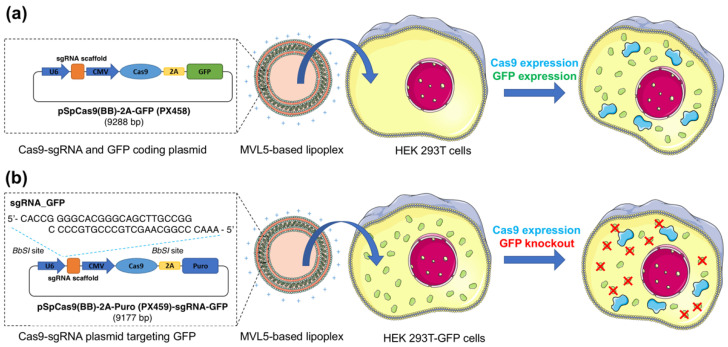
The experimental approach to study Cas9/sgRNA plasmid transfection and gene knockout. (**a**) A plasmid encoding both Cas9 and GFP cassettes is encapsulated into multivalent cationic lipid lipoplexes and administered to HEK 293T cells in vitro. The emergence of a green fluorescence signal in the cells, which indirectly indicates Cas9 expression, is detected by flow cytometry and fluorescence microscopy. (**b**) To measure CRISPR-mediated gene knockout, HEK 293T cells stably expressing GFP are used. A Cas9 expression plasmid containing a sgRNA to target the *GFP* gene (PX459-sgRNA-GFP) is designed. The depletion of fluorescence signal associated with GFP knockout is detected by flow cytometry and fluorescence microscopy. To distinguish between CRISPR-mediated GFP knockout and non-specific GFP reduction (e.g., caused by cytotoxicity), a similar plasmid without the sgRNA targeting sequence is used. U6: U6 promoter; sgRNA: contains a target sequence; CMV: CMV promoter, Cas9: Cas9 expression cassette; 2A: 2A self-cleaving peptide; GFP: GFP selection marker; Puro: Puromycin selection marker.

To measure the TE of the CRISPR/Cas9 plasmid, the plasmid that fuses the reporter green fluorescence protein (GFP) gene and the Cas9 expression cassette (pSpCas9(BB)-2A-GFP (PX458)) was used to facilitate the detection of Cas9 expression in the transfected cells (Figure 1a). Moreover, a Cas9 expression plasmid containing a sgRNA to target the *GFP* gene (PX459-sgRNA-GFP) was constructed (Figure 1b) to evaluate the gene knockout efficiency, being the knockout efficiency determined by the loss of GFP signal. To account for the loss of GFP signal caused by toxicity or non-specific knockout, the PX459 empty vector was also used as a control. 

## 2. Materials and Methods

### 2.1. Reagents and Materials

Plasmids pSpCas9(BB)-2A-Puro (PX459) V2.0 (Addgene plasmid #62988) and pSpCas9(BB)-2A-GFP (PX458) (Addgene plasmid #48138) were a gift from Feng Zhang [59]. Endotoxin-free plasmids were extracted using ZymoPURE II Plasmid Maxiprep Kit from Zymo Research. Lipofectamine™3000, Texas-Red DHPE, and LysoSensor Green DND-189 were purchased from ThermoFisher Scientific. 3-(4,5-dimethylthiazole-2-yl)-2,5-diphenyl-2H-tetrazolium bromide (MTT) and paraformaldehyde and dimethyl sulfoxide (DMSO) were purchased from Sigma. 4′,6-diamidino-2-phenylindole (DAPI) was purchased from Biotium. All cell culture reagents were purchased from Biochrom. Venor™ GeM Mycoplasma Detection Kit was purchased from Merck. The gene target sequences were synthesized by Alfagene. Lipids MVL5, DOTAP, DOPC, and DOPE were purchased from Avanti Polar Lipids (USA). GMO was purchased from Nu-Chek Prep (Elysian, MN, USA). All lipids were used as received. F-Luc-GFP lentivirus was purchased from Capital Biosciences.

### 2.2. Liposome Preparation 

**Figure 2:**
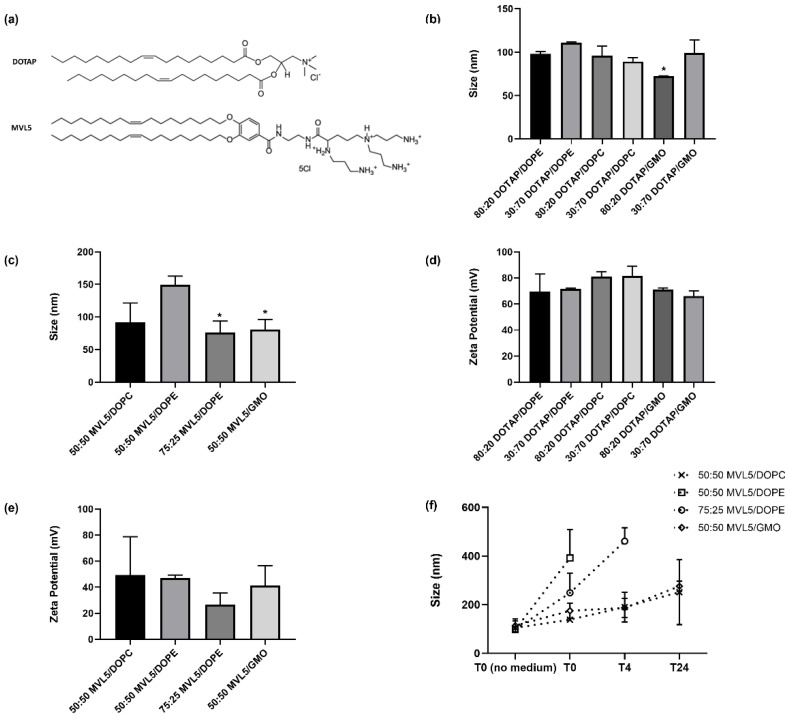
Chemical structure of monovalent cationic lipid DOTAP and multivalent cationic lipid MVL5 (**a**). Biophysical characterization of monovalent (DOTAP), and multivalent (MVL5) lipoplexes incorporating the PX458 plasmid at CR (+/−) 3 (**b**–**d**) and CR (+/−) 10 (**e**). The average size and zeta potential of DOTAP and MVL5-based lipoplexes were measured in ultrapure nuclease-free Milli-Q water. Data represent at least three independent experiments and are presented as the mean ± SD. Formulations marked with an asterisk showed bimodal distributions fitted with a biexponential decay model. (**f**) DLS measurements of the hydrodynamic diameter of the MVL5-based lipoplexes incubated with DMEM at 37 °C. Formulations marked with “*” were fitted with a biexponential decay model.

Liposomes were prepared with different lipid compositions and membrane charge densities (σ_M_). To achieve this, cationic DOTAP or MVL5 (Figure 2a) were mixed with different helper lipids DOPC, DOPE, or GMO at different molar fractions. Lipid stocks dissolved in chloroform were mixed in the desired ratios. For cellular uptake and intracellular distribution studies, liposomes included also 0.1 mol% of total lipid of Texas-Red-DHPE. The resulting mixture was dried using a constant nitrogen gas stream and then placed in a vacuum overnight. The lipid film was resuspended in ultrapure nuclease-free Milli-Q water. The suspensions were vortexed and sonicated using a tip sonicator for 1 min, with 10% amplitude and 50% duty cycle using a Branson Digital Sonifier 250 Model. 

### 2.3. Lipoplex Preparation and Characterization

For lipoplex formation, equal volumes of liposomes and DNA solutions were mixed to the desired concentration. Lipoplexes were prepared with a cationic-to-anionic CR (+/−) of 3 and 10. The CR (+/−) is calculated as the total number of positive charges (from the number and valence of DOTAP or MVL5 molecules) divided by the total number of negative charges (from the number and valence of DNA molecules). The nominal charge of +5*e* was assumed for MVL5, although experimental data at near-physiological conditions indicate that the average charge is closer to +4.5*e* [33]. The resulting mixtures were promptly vortexed for 30 s and left at least 30 min under stirring conditions. The formed complexes were stored at 4 °C. The sizes and zeta potential of the liposome solutions were determined with Dynamic Light Scattering (DLS), using an SZ-100 device from Horiba, measuring scattering at a detection angle of 173°. The autocorrelation (AC) function is fitted using the cumulants method, which provides the diffusion coefficient of the particles and respective polydispersity index (PDI) [60]. In some cases, samples showed AC curves evidencing two size populations. In such cases, the AC curves were fitted with a biexponential decay model, providing the diffusion coefficient of both populations. The particle size (hydrodynamic diameter) is then obtained through the Stokes-Einstein relation. Each sample was measured for three runs of 60 s.

The stability of the MVL5-based lipoplexes was assessed by DLS measurements of the hydrodynamic diameter of the multivalent CL-DNA complexes incubated with cell-cultured medium (DMEM) at 37 °C for 24 h. 

### 2.4. Cell Culture

The human embryonic kidney (HEK) 293T (ATCC CRL-3216) cell line and HEK 293T cell line with stable GFP expression were cultured in Dulbecco’s minimal essential medium (DMEM), supplemented with 10% heat-inactivated fetal bovine serum (FBS) and 1% penicillin-streptomycin. Cells were grown in polystyrene tissue culture flasks in a humidified atmosphere of 5% CO_2_ and 37 °C and subcultured using 0.25% Trypsin-EDTA solution. Mycoplasma testing by PCR was carried out routinely using Venor™ GeM Mycoplasma Detection Kit.

HEK293T stably expressing GFP (HEK293T-GFP) cell line was generated by transduction with F-Luc-GFP lentivirus in which GFP was expressed under the puromycin resistance marker. The infection was carried out at a multiplicity of infection (MOI) of 10 in a complete medium supplemented with 5 µg/mL of Polybrene. Stably transduced cells were selected by adding 2 µg/mL of Puromycin, and the selection was conducted for 14 days.

### 2.5. In Vitro Transfection and Gene Expression Analysis

To evaluate in vitro transfection, HEK 293T cells were transfected with the pSpCas9(BB)-2A-GFP (PX458) plasmid, which contains both Cas9/sgRNA and GFP expression cassettes. Cells were plated at a density of 2 × 10^5^ cells/well in a 6-well plate and grown to approximately 60–70% confluency before transfection. All CL-DNA complexes, containing 1 or 2 µg of PX458, were diluted to a final volume of 1 mL in DMEM medium (in absence of serum) and transferred onto cells. Then, 4 h post-transfection, the complexes were removed, and the medium was replaced by a complete DMEM medium for an additional 48 h of incubation. Lipofectamine^®^3000-DNA complexes were used as a positive control according to the manufacturer’s instructions. *GFP* gene expression was measured on an EC800 Flow Cytometry Analyzer (Sony Biotechnology Inc., San Jose, CA, USA) by counting at least 20,000 events. Analysis of data was performed on the FlowJo 10.8.0 software to calculate the percentage of GFP-positive cells. In addition to flow cytometry, GFP-expressing cells were also visualized by fluorescence microscopy. For this, cells transfected beforehand were fixed with 4% paraformaldehyde for 40 min at room temperature, followed by counterstaining with DAPI for 15 min at room temperature. Cells were observed in a fluorescence microscope [OLYMPUS BX51] incorporated with a high-sensitivity camera Olympus DP71 at 10× magnification. Images were analyzed by ImageJ (Version 1.51q, National Insitutes of Health, Bethesda, MD, USA).

### 2.6. In Vitro Cytotoxicity Assay

The colorimetric MTT assay was used to evaluate the effect of CL-DNA complexes incorporating Cas9/sgRNA plasmid on cell viability. Then, 1 × 10^4^ HEK 293T cells were plated on 96-well culture plates and incubated overnight. Then, cells were incubated with CL-DNA complexes containing 0.1 µg DNA per well for 4 h. After incubation, the CL-DNA complex solution was replaced by DMEM complete medium. Cell viability was measured after 48 h by adding to each well 0.5 mg/mL of MTT and incubating for 4 h at 37 °C. The blue formazan crystals formed by viable cells were dissolved in DMSO, and their optical density was assessed at a wavelength of 570 nm in a microplate reader (Cytation 3, BioTek, Winooski, VT, USA).

### 2.7. Cellular Uptake and Intracellular Distribution

To observe cellular uptake and intracellular distribution of cationic liposomes incorporating CRISPR/Cas9 DNA plasmids, monovalent and multivalent cationic liposomes at CR (+/−) 3 were prepared using Texas-Red-labelled liposomes, as described above. The CL-DNA complexes were formulated with CRISPR/DNA plasmid (PX458) and added to HEK 293T cells previously seeded on coverslips in a 24-well plate (5 × 10^4^ cells/well) at a final concentration of 2 µg/mL of DNA. The transfected cells were incubated for 4 h at 37 °C, and then 1 µM of LysoSensor Green DND-189 was added to each sample to label lysosomes and sustained for 30 min at 37 °C. Next, the medium was removed, and the coverslips were observed using a 60× and 100× oil immersion objective in a fluorescence microscope [OLYMPUS BX51] incorporated with a high-sensitivity camera Olympus DP71. Images were analyzed by ImageJ software.

### 2.8. GFP Gene Disruption Assay

A single-guide RNA (sgRNA) was selected to target GFP sequence (sgRNA-GFP: GGGCACGGGCAGCTTGCCGG). The sgRNA was inserted into the *BbSI* sites of pSpCas9(BB)-2A-Puro (PX459) plasmid. The multivalent CL-DNA complexes were formed incorporating 2 µg of PX459-sgRNA-GFP (Figure 1b), and then transferred onto HEK 293T-GFP cells, seeded beforehand on a 6-well plate at 2 × 10^5^ cells/well. Cells were exposed to CL-DNA complexes for 4 h in a free-serum medium, and then the CL-DNA complex solution was replaced by DMEM complete medium. The unmodified PX459 plasmid was used as a negative control, and Lipofectamine^®^3000-DNA complexes were used as a positive control in *GFP* gene disruption. GFP knockout was assessed 72 h after transfection by the percentage of GFP-negative cells, evaluated using EC800 Flow Cytometry Analyzer (Sony Biotechnology Inc., San Jose, CA, USA). A total of 15,000 events were counted. 

### 2.9. Statistical Analysis 

Data were expressed as mean ± standard deviation (SD) of at least two independent experiments. One-way ANOVA with Dunnett’s multiple comparisons test and two-way ANOVA with Sidak’s multiple comparisons test were performed using GraphPad Prism 8.3.0 (GraphPad Software, Inc., San Diego, CA, USA) to identify differences among multiple groups, considering a significance level of 95%.

## 3. Results and Discussion

### 3.1. Lipoplex Size and Zeta Potential

Figure 2b,c shows the average particle sizes of the different formulations used. For the DOTAP/helper lipid mixtures two molar ratios were used, 80:20 and 30:70, respectively. Most particles have sizes between 85–120 nm, with the exception of the molar ratio of 80:20 in the DOTAP/GMO mixture, which shows a bimodal distribution in all three replicas (a population of particles with a size of 73 ± 1 nm, which represents most of the particles and is shown on the figure, plus a small population of >20 μm aggregates). Regarding the MVL5 formulations, the cationic to neutral lipid molar ratio was 50:50, with the additional formulation of MVL5/DOPE at 75:25. In contrast to DOTAP, the MVL5 formulations have more distinct sizes among themselves. The 50:50 MVL5/DOPE particles are the largest, with sizes of 149 ± 11 nm. The 75:25 MVL5:DOPE showed the smallest sizes (76 ± 14 nm), although one of the replicas exhibited a slight amount of a second population with >20 μm particles. The MVL5/GMO particles, such as the ones in DOTAP/GMO 80:20, indicated the presence of two populations in two of the replicas, with the dominant population (shown in the figure) with a size of 81 ± 13 nm, and a small population of >20 μm particles (Table S1).

The zeta potential of the CL-DNA complexes was also assessed. Both DOTAP and MVL5-based lipoplex formulations are positively charged (Figure 2d,e), with the DOTAP-based formulations exhibiting larger zeta potential values. The DOTAP/DOPC mixtures exhibited the highest potential, corresponding to 82 ± 6 mV and 81 ± 3 mV for the molar ratio of 30:70 and 80:20, respectively. Regarding the MVL5-based lipoplexes, all formulations showed similar values of potential (around 40–50 mV), except for the 75:25 MVL5:DOPE mixture, which displayed the lowest value (26.5 ± 9.1 mV). 

**Figure 3:**
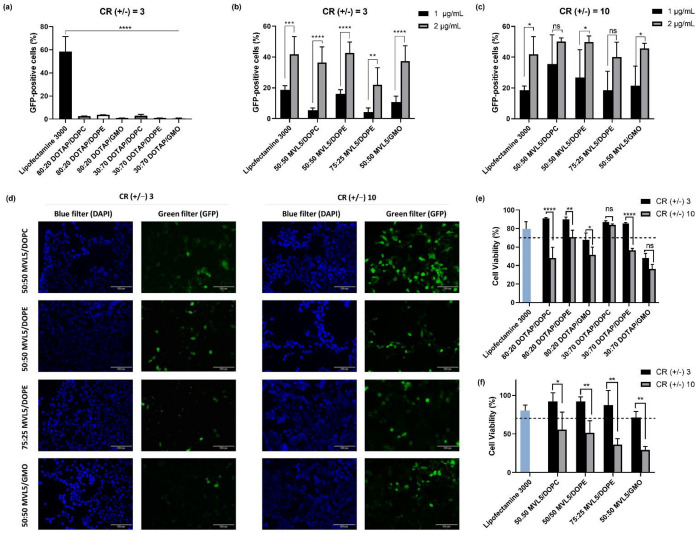
*In vitro* transfection and cytotoxicity in HEK 293T cells transfected with the Cas9/sgRNA-GFP plasmid (PX458). The percentage of GFP-positive cells was measured by flow cytometry. The data are expressed as the mean ± SD (*n* = 3). (**a**) Transfection efficiency of DOTAP-based lipoplexes at CR (+/−) 3. One-way ANOVA indicates statistically significant differences within the group assessed by Dunnett’s multiple comparisons test and denoted as follows: **** *p* ≤ 0.0001. (**b**,**c**) Transfection efficiency of MVL5-based lipoplexes at a CR (+/−) of 3 and 10, respectively. (**d**) Fluorescence microscopy images of HEK 293T cells transfected with 2 µg/mL of PX458 plasmid via multivalent CL-DNA complexes. Scale bars correspond to 100 µm. (**e**,**f**) Cytotoxicity profile of monovalent (DOTAP) and multivalent (MVL5) lipoplexes, respectively, as evaluated by the MTT assay. HEK 293T cells were transfected with lipoplexes containing 0.1 µg of PX458. Untreated cells were used as a negative control (100% viable cells). The dashed line corresponds to 70% of cell viability. Data are expressed as the mean ± SD (*n* = 3). Two-way ANOVA indicates statistically significant differences within the group assessed by Sidak’s multiple comparisons test and denoted as follows: **** *p* < 0.0001, *** *p* < 0.001, ** *p* < 0.01, * *p* < 0.1, and ns *p* > 0.05.

The stability of the multivalent lipoplexes was evaluated by incubating these with a cell culture medium (DMEM) at 37 °C. Figure 3f shows the hydrodynamic diameter of the CL-DNA complexes prior to the medium addition (T0 no medium) and after the dilution in DMEM at three time points (0, 4, and 24 h). The hydrodynamic diameter of the DOPC and GMO complexes showed a small increase in size immediately after the medium change, and a moderate increase during the following 24 h. This indicates suitable colloidal stability of these CRISPR delivery systems. In contrast, the MVL5/DOPE formulations increased their size to more than twice already at T0 and continued to increase over time until strong aggregation was observed, making it not possible to measure their size at the 24 h time point.

### 3.2. In Vitro Transfection 

To evaluate the in vitro transfection, HEK 293T cells were exposed for 4 h to monovalent or multivalent CL-DNA complexes encapsulating the Cas9/sgRNA-GFP plasmid (PX458). This plasmid design facilitates the detection of positively transfected cells through the expression of GFP (Figure 1a). The number of GFP-positive cells was determined after 48 h of incubation by flow cytometry. Lipofectamine 3000, the most potent commercially available in vitro transfection reagent, was chosen as the positive control. Figure 3a shows the percentage of GFP-expressing cells after treatment with monovalent lipoplexes with CR (+/−) fixed at three and different cationic to neutral lipid ratios (30:70 and 80:20). This CR (+/−) of three was found to be optimal for transfection of mouse L-cells with monovalent cationic lipid lipoplexes [61]. However, HEK 293T cells treated with DOTAP-based lipoplexes at a final DNA concentration of 2 µg/mL showed a very low percentage of GFP-positive cells in comparison to the commercial transfection reagent (*p* < 0.0001). Even though HEK 293T cells are not particularly easy to transfect with monovalent cationic lipoplexes, we were expecting a measurable improvement with the 80:20 lipoplexes, as evidenced by transfection with luciferase reporter genes [62]. This lack of improvement suggests that the larger size of the Cas9 plasmid, which is almost twice the size of the luciferase reporter genes, makes it harder to transfect cells efficiently, and demonstrates that these formulations are not able to improve gene delivery for CRISPR/Cas9-based applications.

In stark contrast, the MVL5-based lipoplexes (Figure 3b,c and Figure S1) showed meaningful levels of transfection for all the tested formulations, reaching values such as those with lipofectamine in some cases. This may be caused by the significantly higher lipid membrane charge density of the multivalent complexes, which has been suggested to facilitate the escape of the lipoplexes to the cytosol through the fusion of the lipid and endosomal membranes [32].

Interestingly, the increase of DNA concentration from 1 to 2 μg/mL produced a strong increase in the TE, especially at CR (+/−) of 3. Increasing the CR (+/−) from 3 to 10 also improved the TE, but this effect was much more visible for the 1 μg/mL CL-DNA complexes. For the 2 μg/mL CL-DNA solutions, the increase in CR (+/−) produced a milder improvement in the TE. The milder improvement at CR (+/−) 10 may be the result of a much higher concentration of cationic lipid in the cells, which may, in turn, result in some additional cytotoxicity that hampers the TE, as discussed below. 

Regarding the type of neutral lipid used, the MVL5-based formulations exhibited similar transfection efficiencies under the same conditions, which may be an indication that the interactions of these complexes with cells, and ensuing TE, are dominated by the strong charge emanating from the multivalent cationic lipids, and that the type of neutral lipid, whereas it is more fusogenic or not, plays a weaker role. This is not entirely surprising, since these lipoplex compositions are expected to have similar internal structures of the multilamellar type, hence, favoring similar mechanisms of interaction with cells.

In addition to the flow cytometry results, transfection by MVL5 lipoplexes at 2 μg/mL DNA was also analyzed by fluorescence microscopy imaging (Figure 3d). Such results show bright green fluorescence signals resulting from GFP-expressing cells, being more evident for CR (+/−) 10 lipoplexes and corroborating the flow cytometry results. Both flow cytometry and fluorescence microscopy data demonstrate that multivalent CL-DNA complexes at CR (+/−) 10 are effective vectors for plasmid-based CRISPR/Cas9 systems in vitro, encouraging further investigation in its potential translation for in vivo applications. 

While the two-component lipid formulations based on DOTAP were shown to have a poor performance, recent studies have successfully demonstrated the delivery of CRISPR/Cas9 systems using more complex DOTAP-based lipoplexes [63,64,65]. For instance, Hosseini et al. [64] showed that the DOTAP/DOPE/Chol-Polyethylene Glycol system can successfully transfect the Cas9/sgRNA plasmid into HEK 293 cells stably expressing GFP, leading to a *GFP* gene knockout of 39%. This achievement may be partly explained by the incorporation of cholesterol, which is known to improve the TE in monovalent CL-DNA complexes [66] and some studies have demonstrated its important role in intracellular trafficking [67,68,69]. In the present work, we showed that replacing DOTAP with MVL5 in simple two-lipid formulations leads to a pronounced improvement in the transfection of Cas9/sgRNA plasmids. By further optimization of the multivalent cationic lipid formulations, i.e., by adjusting the DNA concentration and CR (+/−), as well as incorporating additional lipids such as cholesterol, these formulations may become highly efficient and compete with viral-delivery methods.

### 3.3. Cytotoxicity of Monovalent and Multivalent CL-DNA Complexes 

The evaluation of cell viability impact is particularly relevant for the development of safe and effective gene delivery systems because cytotoxicity influences the transfection rate efficiency. Cytotoxicity of CL-DNA complexes was assessed using the standard colorimetric MTT assay. HEK 293T cells were transfected by CL-DNA complexes for 48 h, and untreated cells were used as a positive control and normalized to 100% cell viability. According to ISO 10993-5, no cytotoxic effect is considered in cell viability for values greater than 70%. As shown in Figure 3e, monovalent CL-DNA complexes at CR (+/−) 3 demonstrated biosafety, except for 80:20 and 30:70 DOTAP/GMO formulations, which exhibited a survival rate of 67.9 ± 6.6% and 48.1 ± 4.5%, respectively. Regarding the complexes at CR (+/−) of 10, there was a significant decrease of cell viability for nearly all the formulations compared to those at lower CR (+/−) after 48 h of transfection. The 80:20 DOTAP/DOPC and 30:70 DOTAP/DOPE formulations exhibited the most significant increase in toxicity (*p* < 0.0001), followed by 80:20 DOTAP/DOPE (*p* < 0.001) and 80:20 DOTAP/GMO (*p* < 0.01). 

Concerning multivalent CL-DNA complexes (Figure 3f), the same trend was observed. All multivalent CL-DNA complexes at CR (+/−) 3 were demonstrated to be safe enough for gene delivery since no obvious cytotoxic impact was observed on HEK 293T transfected cells. The average cell viability of CR (+/−) 3 formulations (86%) was significantly superior to those prepared at CR (+/−) 10 (57%). Moreover, both monovalent and multivalent cationic systems at CR (+/−) 3 exhibited less cytotoxicity than the commercial transfection reagent Lipofectamine 3000. These results suggest that the CR (+/−) influences the viability of the transfected cells. This is not surprising since for a fixed DNA concentration, increasing the CR (+/−) results in an increase in the lipid concentration used, and therefore, in an increase in cytotoxicity. 

Overall, the observed interdependence between the DNA concentration and CR (+/−) on the TE (Figure 3b,c) suggests that by careful tuning of these two parameters, the TE and cytotoxicity can be adjusted to an effective and safe CRISPR/Cas9 delivery system.

### 3.4. Cellular Uptake and Intracellular Distribution of Monovalent and Multivalent CL-DNA Complexes

**Figure 4:**
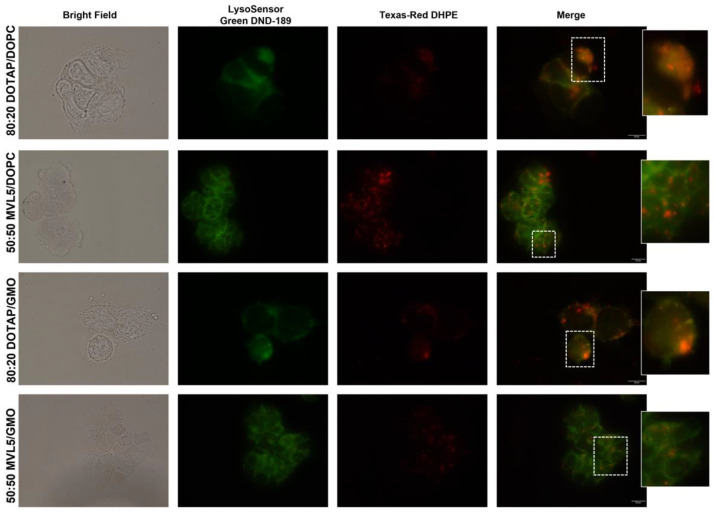
Colocalization of DOTAP and MVL5 lipoplexes signal (red) with LysoSensor (lysosome marker, green) after 4 h of transfection. HEK 293T cells were transfected with Texas-Red-DHPE labeled lipoplexes for 4 h at 37 °C, and then stained with LysoSensor to track the lysosomes’ location. Merged files are representative of the colocalization of CL-DNA complexes with lysosomes. Images were obtained by fluorescence microscopy using a 60× and 100× immersion oil objective. Scales bar corresponds to 10 and 20 µm.

To understand the TE differences exhibited by monovalent and multivalent CL-DNA complexes, cellular uptake and intracellular distribution studies of these lipoplexes were performed. The TE of lipoplexes greatly depends on their ability to overcome intracellular barriers to deliver exogenous DNA into the cell nucleus of the host cell and enable its expression. The cellular uptake mechanism and trafficking to lysosomes are critical for efficient delivery since the rearrangement of lipoplexes structure during those stages influences the DNA escape process and release into the cytoplasm [70]. Therefore, colocalization analysis of fluorescence signals from labeled lipoplexes (red, Texas-Red-DHPE) and lysosomes (green, LysoSensor Green DND-189) was performed to evaluate the intracellular fate of lipoplexes. Despite both monovalent and multivalent lipoplexes being uptaken by HEK 293T cells, DOTAP-based lipoplexes were predominantly found in the lysosomes originating a yellowish signal derived from the colocalization of lipoplexes and lysosomes (Figure 4). This observation suggests that DOTAP-based lipoplexes have a poor endosomal release capacity in HEK 293T cells, eventually undergoing lysosomal degradation, which results in low TE. In contrast, the absence of colocalization of the MVL5-based lipoplexes with LysoSensor suggests that these formulations can elude metabolic degradation and escape from lysosomal entrapment. This remark might explain the superior TE of MVL5-based lipoplexes over monovalent lipoplexes, highlighting the potential of MVL5 to boost the TE of lipid-DNA complexes. Interestingly, no differences were obvious between using DOPC or GMO as the helper lipid, which as pointed out above, hints that fusion of the lipoplex membranes with the endosomes and subsequent endosomal escape is dominated by the high membrane charge density imposed by MVL5. 

### 3.5. GFP Disruption Mediated by Multivalent CL-DNA Complexes

**Figure 5:**
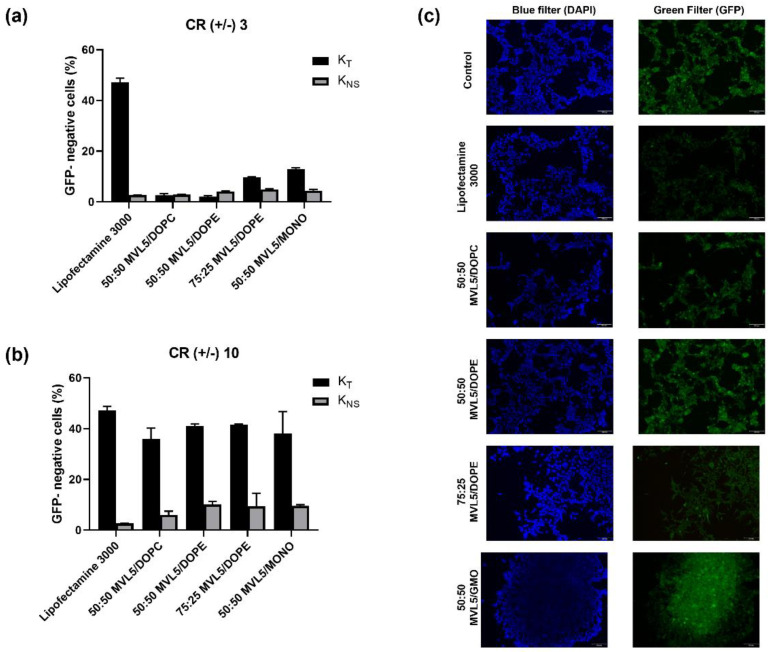
*GFP* gene disruption assay in HEK 293T-GFP cells, measured 72 h after treatment with CL-DNA complexes and Lipofectamine 3000. (**a**,**b**) Total (K_T_) and non-specific (K_NS_) gene silencing efficiency of MVL5-based lipoplexes at a CR (+/−) of 3 and 10, respectively. The percentage of GFP-negative cells was measured by flow cytometry. The data are expressed as the mean± SD (*n* = 2). K_T_ is measured with the PX459-sgRNA-GFP and K_NS_ is measured with the PX459 empty vector. (**c**) Fluorescence microscopy images of HEK 293T-GFP cells transfected with MVL5-lipoplexes at a CR (+/−) of 10. Scales bars correspond to 100 µm.

To evaluate the potential of MVL5-lipoplexes to deliver Cas9/sgRNA plasmids and induce gene disruption in vitro, HEK 293T-GFP cells were used, and a plasmid encoding the Cas9 protein and sgRNA targeting the *GFP* gene (PX459-sgRNA-GFP) was designed (Figure 1b). The *GFP* gene disruption efficiency was quantitatively determined with flow cytometry by measuring the decrease in the number of green fluorescence positive cells. The PX459 empty vector was used as a negative control to assess any non-specific effect of the vector/backbone itself on *GFP* gene expression in HEK293T-GFP cells. The loss of GFP signal resulting from the PX459-sgRNA-GFP and PX459 plasmid transfection are plotted as total knockout (K_T_) and non-specific gene knockout (K_NS_), respectively (Figure 5 and Figure S2).

As shown in Figure 5a, for CR (+/−) of 3, the K_T_ of MVL5 lipoplexes is relatively low when compared to that of lipofectamine, but it increases substantially when the CR (+/−) is increased to 10 (Figure 5b). However, the non-specific gene knockout (K_NS_) is also more pronounced in those formulations. These expressive values of K_NS_ could be explained by the toxic effects caused by these formulations at CR (+/−) 10, which are particularly high for the MVL5/DOPE 75:25 and MVL5/GMO 50:50 as previously demonstrated (Figure 3f). The discrepancy between transfection and knockout results from MVL5-based complexes when compared to lipofectamine could be associated with the higher toxicity observed for the former, that is, cells incubated with MVL5-lipoplexes are still able to express Cas9, but the subsequent gene knockout processes are hampered by the formulations cytotoxicity. Yet, all MVL5-based formulations at CR (+/−) 10 show a total K_T_ superior to 35%.

GFP expression was also observed by fluorescence microscopy (Figure 5c). Analyzing the images, it is possible to observe that the GFP signal is weaker in cells transfected with Lipofectamine 3000 in comparison to MVL5-based lipoplexes, which is in agreement with the flow cytometry results. 

Overall, these results show that MVL5-based lipoplexes enabled the delivery of Cas9/sgRNA plasmids to human epithelial kidney cells and mediated GFP knockout via the CRISPR/Cas9 system at levels comparable with the commercial transfection reagent Lipofectamine 3000^®^. The knockout efficiency was especially high for CR (+/−) 10, although cytotoxicity from the formulations was also significant and may have contributed to a significant level of non-specific gene silencing. Nevertheless, these MVL5-based formulations can still be optimized by further adjusting the CR (+/−) and DNA concentration, which can lead to a better cytotoxic profile while keeping a suitable TE and gene knockout efficiency. They can also be modified to include additional lipids (e.g., cholesterol) or surface modifications (e.g., PEGylation and targeting ligands) to improve transfection and knockout efficiencies in vitro and in vivo even further, which is difficult to achieve with the available commercial transfection reagents.

## 4. Conclusions

In this work, we investigated the suitability of simple multivalent cationic-DNA complexes to deliver a Cas9/sgRNA expressing plasmid for genome editing. The liposomes used are composed of two lipids, one cationic and one helper lipid. Whereas all the monovalent DOTAP-based formulations resulted in extremely inefficient transfection, MVL5-based formulations exhibited both high transfection efficiency as well as gene knockout ability for all the helper lipids used. In addition, MVL5-based lipoplexes were also found to have lower colocalization with lysosomes, which suggests enhanced endosomal release when compared with DOTAP formulations. Overall, this suggests that the higher membrane charge density imposed by MVL5 is the main factor contributing to fusion with the endosomal membrane and consequent enhancement of transfection efficiency. Despite the transfection efficiency rates being comparable to lipofectamine 3000^®^ commercial reagent, the *GFP* gene knockout was demonstrated to be slightly inferior. One drawback is that the MVL5-based formulations also showed considerable non-specific gene knockout, probably resulting from their higher cytotoxicity. Nevertheless, the versatility of these formulations provides significant opportunities for further optimization, either by tuning the cationic-to-anionic CR (+/−) to lower cytotoxicity or by including additional lipids or surface functionalization, which is important for in vivo applications. These results show that multivalent lipid-based lipoplexes are promising CRISPR/Cas9 plasmid delivery systems, and by further optimization and functionalization may constitute an alternative to viral-delivery methods and to ionizable lipid mRNA-based delivery lipid nanoparticles.

## Data Availability

Not applicable.

## Supplementary Materials

The following supporting information can be downloaded at: https://www.mdpi.com/article/10.3390/pharmaceutics14051087/s1, Table S1: Biophysical characterization of monovalent (DOTAP) and multivalent (MVL5) lipoplexes; Figure S1: Flow cytometry of HEK293T cells transfected with the Cas9/sgRNA-GFP plasmid (PX458); Figure S2: Flow cytometry histograms illustrating the GFP signal in HEK 293T-GFP cells.

## Abbreviations

AAV	Adeno-associated virus (AAV)
Cas9	CRISPR-associated nuclease 9
CLs	Cationic liposomes
CR	Charge ratio
CRISPR	Clustered regularly interspaced short palindromic repeats
DAPI	4′,6-diamidino-2-phenylindole
DLS	Dynamic light scattering
DMSO	Dimethyl sulfoxide
DOPC	Dioleoylphosphocholine
DOPE	Dioleoylphosphatidylethanolamine
DOTAP	2,3-Dioleyloxypropyltrimethylammonium chloride
DNA	Deoxyribonucleic acid
GFP	Green fluorescence protein
GMO	Glycerol-monooleate
HDR	Homology directed repair
HEK	Human embryonic kidney
K_NS_	Non-specific knockout
K_T_	Total knockout
mRNA	messenger RNA
MTT	3-(4,5-dimethylthiazole-2-yl)-2,5-diphenyl-2H-tetrazolium bromide
NJEH	Nonhomologous end-joining
Puro	Puromycin
PCR	Polymerase chain reaction
RNA	Ribonucleic acid
SD	Standard deviation
sgRNA	Single-guide RNA
siRNA	Small interfering RNA
TE	Transfection efficiency
