# Supplementary material for: In Vitro CRISPR/Cas9 Transfection and Gene-Editing Mediated by Multivalent Cationic Liposome–DNA Complexes
Source: Pharmaceutics. 2022 May 19;14(5):1087. doi: 10.3390/pharmaceutics14051087 (PMC9143451; doi:10.3390/pharmaceutics14051087)
Supplement: Supplementary file 1 [file pharmaceutics-14-01087-s001.zip › pharmaceutics-1668578-supplementary.pdf]

pharmaceutics

Supplementary Materials: In vitro CRISPR/Cas9 transfection and gene-editing mediated by Multivalent Cationic Liposome-DNA complexes

Diana A. Sousa, Ricardo Gaspar, Celso Ferreira, Fátima Baltazar, Ligia R. Rodrigues, and Bruno F.B. Silva

Supplementary Files

Table S1. Biophysical characterization of monovalent (DOTAP) **(a)**, and multivalent (MVL5) **(b)** lipoplexes incorporating the PX458 plasmid at CR (+/-) 3 and CR (+/-) 10 **(c)** , respectively. The average size and zeta potential of DOTAP and MVL5-based lipoplexes were measured in ultrapure nuclease free Milli-Q water. Standard deviation (std) and Polydispersity Index (PDI) are also presented. Formulations marked with "*" or "**" showed bimodal or monomodal distributions fitted with a biexponential or monoexponential decay models, respectively.

**(a)**						
**CR (+/-) 3**	Size (nm)	std	PDI	std	ZETA (mV)	std
80:20 DOTAP/DOPE	98.0	2.2	0.03	0.01	69.6	11.0
30:70 DOTAP/DOPE	111.0	0.8	0.20	0.02	71.7	0.5
80:20 DOTAP/DOPC	96.0	9.3	0.13	0.07	81.1	3.1
30:70 DOTAP/DOPC	89.3	3.8	0.22	0.02	81.7	6.1
80:20 DOTAP/GMO*	66.3	1.2	Bi-Exp	-	71.2	0.9
30:70 DOTAP/GMO**	99.0	12.2	Mono	-	66.0	3.3
**(b)**						
**CR(+/-) 3**	Size (nm)	std	PDI	std	ZETA (mV)	std
50:50 MVL5/DOPC	92.0	25.7	0.25	0.08	64.0	46.4
50:50 MVL5/DOPE	149.0	11.2	0.23	0.21	10.86	20.2
75:25 MVL5/DOPE**	76.0	14.3	Bi-Exp	-	12.94	19.6
50:50 MVL5/GMO**	81.0	12.8	Bi-Exp	-	102.34	9.9
**(c)**						
**CR (+/-) 10**	Size (nm)	std	PDI	std	ZETA (mV)	std
50:50 MVL5/DOPC	105.3	7.7	0.3	0.11	49.4	23.9
50:50 MVL5/DOPE	97.2	1.9	0.3	0.02	47.1	6,0
75:25 MVL5/DOPE	118.0	9	0.2	0.08	30.7	12.0
50:50 MVL5/GMO	98.5	4.5	0.3	0.14	46.1	13.1

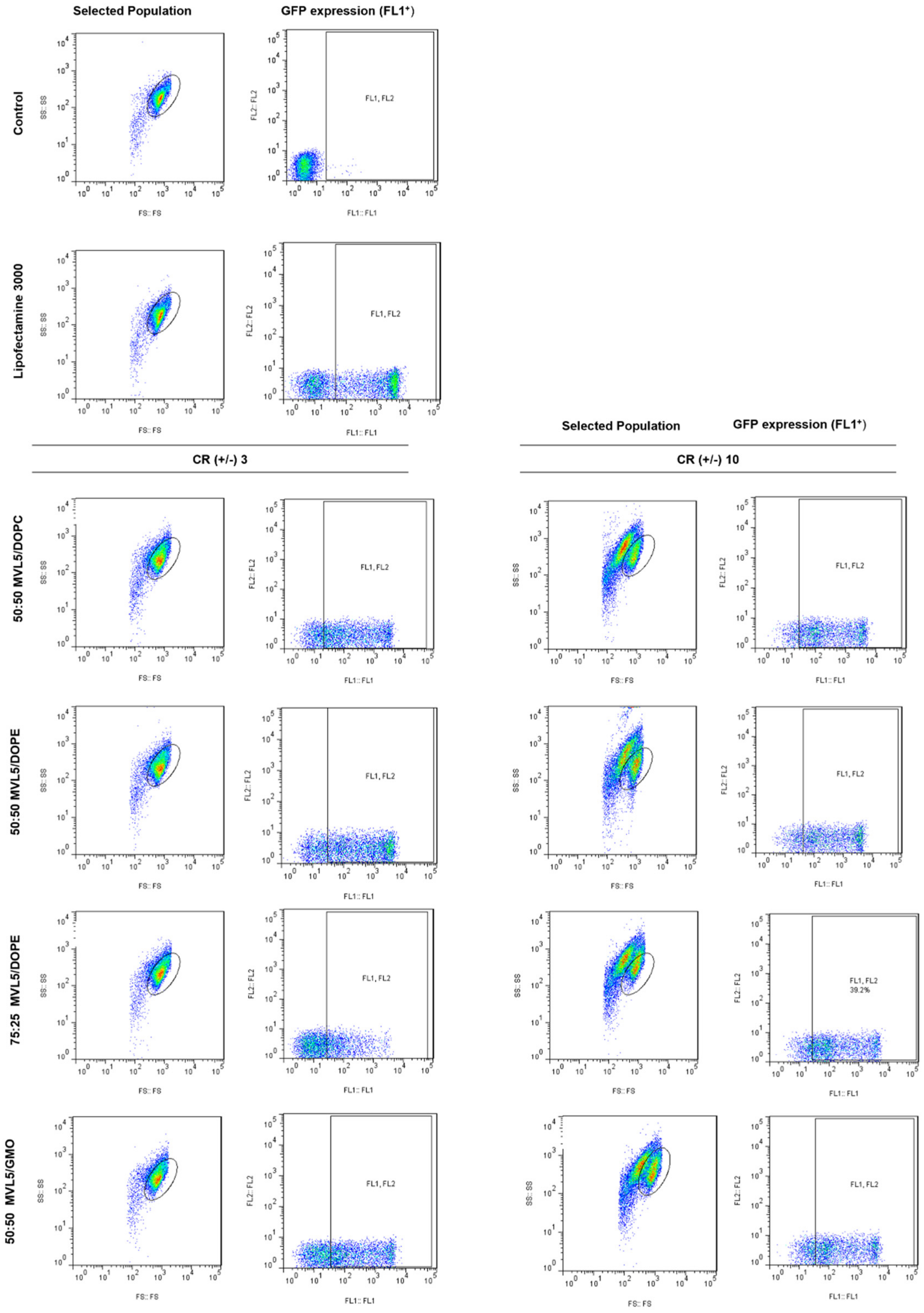

Figure S1. Flow cytometry of HEK 293T cells transfected with the Cas9/sgRNA-GFP plasmid (PX458). The control (HEK 293T untreatead cells) was gated in SS for side-scattered light versus FS for forward-scattered light, and in FL1 versus FL2 for GFP signal). These gates were used for the following fluorescence analysis. Lipofectamine 3000 was used as positive control. FL1, FL2 subset represents GFP-positive cells. GFP was excited by a diode blue laser (488 mm) and the green fluorescence of cells was detected using a 530/50 nm bandpass filter in FL1 channel.

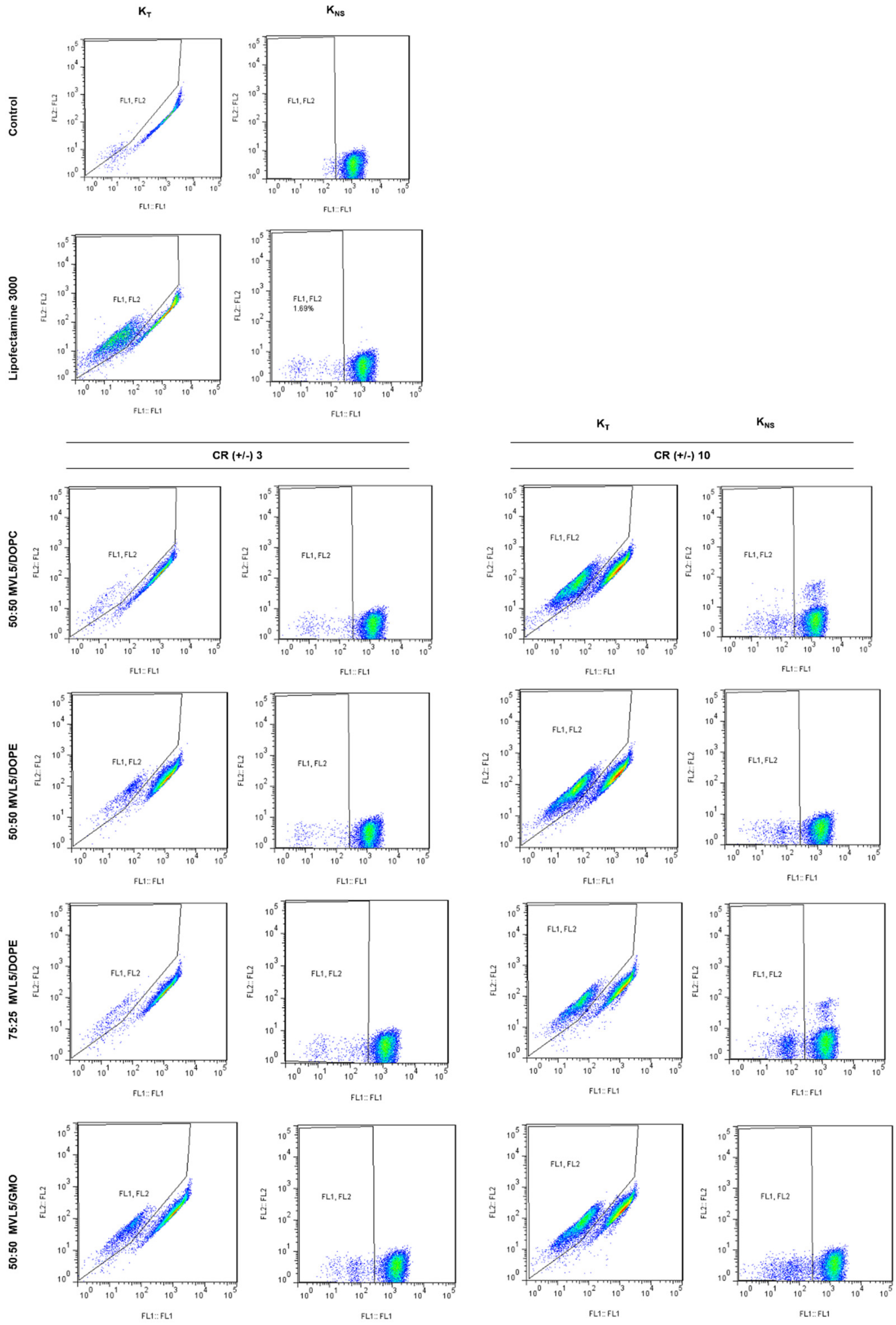

Figure S2. Flow cytometry histograms illustrating the GFP signal in HEK 293T-GFP cells. HEK 293T-GFP cells were treated with CL-DNA complexes containing negative control plasmid (PX459) or PX459-sgRNA-GFP to target *GFP gene*. Lipofectamine 3000 was used as positive control. Cell population and FL1 versus FL2 gates were definied for the control (HEK 293T-GFP cells) and were used for the following fluorescence analysis. FL1, FL2 subset represents the loss of GFP signal. GFP was excited by a diode blue laser (488 mm) and the green fluorescence of cells was detected using a 530/50 nm bandpass filter in FL1 channel. Data are expressed as the mean ± SD (n=2).
